# Fundamental frequency outcomes after type III thyroplasty in cisgender men: a prospective cohort study

**DOI:** 10.1007/s00405-026-10127-w

**Published:** 2026-03-15

**Authors:** Ferhat Alkan, Gamze Yeşilli Puzella, Kürşat Yelken

**Affiliations:** 1 Department of Speech and Language Therapy, Faculty of Health Sciences, Tarsus University, Takbas, Kartaltepe Street, 33400, Türkiye Tarsus/Mersin, Turkey; 2 Department of Speech and Language Therapy, School of Health Sciences, Cappadocia University, Nevşehir, Türkiye Turkey; 3 Voicest Clinic, Istanbul, Türkiye Turkey

**Keywords:** Voice, Pitch, Fundamental frequency, Type III thyroplasty, Voice surgery, Acoustic analysis

## Abstract

**Purpose:**

Persistent high-pitch voice in cisgender men can cause functional and psychosocial burden, and Type III thyroplasty is widely used to lower fundamental frequency (F0). Prospective data with standardized recordings and explicit estimates of F0 change are limited. This study quantified postoperative F0 change and examined whether it depended on elapsed days between recordings.

**Methods:**

In a prospective single-center cohort, cisgender men undergoing Type III thyroplasty completed standardized smartphone recordings of sustained /a/ before surgery and at a single postoperative visit. F0 from a 3-s mid-segment was extracted in Praat. Pre- and postoperative F0 were compared with a paired-samples t-test (ΔF0 = post − pre). Linear regression tested the association between ΔF0 and elapsed days between recordings.

**Results:**

Of 89 patients with a preoperative recording, 33 (37.1%) provided a postoperative sample. Mean F0 decreased from 123.32 Hz (SD 23.76) to 107.58 Hz (SD 19.15); the mean paired difference was ΔF0 = − 15.74 Hz (95% CI − 23.72 to − 7.76, *p* < 0.001). Postoperative recordings occurred a median of 254 days after the preoperative recording (IQR 182–344). ΔF0 was not associated with elapsed days (β = −0.016 Hz/day, *p* = 0.424, R² = 0.020). No intra- or postoperative complications were observed.

**Conclusion:**

Type III thyroplasty in cisgender men yields a moderate, clinically interpretable reduction in F0 that appears robust to variation in postoperative timing. These estimates provide quantitative benchmarks for postoperative counseling and support standardized smartphone recordings in future outcome studies.

## Introduction

The human voice is a primary instrument for communication and self-expression. Acoustic attributes, especially pitch (fundamental frequency, F0), loudness, and resonance shape how listeners perceive a speaker’s age, gender expression, and affective state [1–3]. Additionally, the voice provides cues about the speaker’s gender, age group, and emotional state during verbal communication, and plays a significant role in shaping the listener’s perception of femininity or masculinity [4].

In some cases, individuals may feel uncomfortable with their voices or wish to change their pitch due to reactions from their social environment, even in the absence of any pathology. Research has shown that people adjust their vocal pitch depending on the perceived dominance or social status of their conversational partner [5].

Management of pitch-related complaints ranges from behavioral voice therapy to surgery, selected according to diagnosis and treatment goals [6]. When therapy alone is insufficient or not preferred, Type III (relaxation) thyroplasty can lower F0 while preserving vocal fold vibration and overall voice quality [7–11]. By reducing longitudinal tension through an anterior thyroid cartilage window, the procedure achieves a pitch decrease without impairing vibratory function [8, 10, 11].

Published reports generally document postoperative F0 reductions after Type III thyroplasty. A recent systematic review (9 studies, 69 patients) estimated a pooled mean drop of ~ 76 Hz but emphasized small samples, heterogeneous populations, variable speech tasks, and inconsistent reporting of perioperative therapy and surgical details, underscoring the need for prospective, standardized datasets in clearly defined cohorts [12]. Findings from specific populations (e.g., trans men) likewise show substantial F0 lowering, illustrating how baseline pitch and cohort composition influence effect size [13].

We conducted a prospective, single-center study in cisgender adult men to quantify the change in F0 between preoperative and a single postoperative recording obtained under a standardized protocol. We hypothesized that mean F0 would be lower postoperatively.

## Methods

### Ethical approval and consent

The study was conducted at a tertiary voice clinic in accordance with the Declaration of Helsinki and was approved by the Non-Interventional Clinical Ethics Committee of Cappadocia University (File No: E-64577500-050.99.99-30763), indicating that the study met all ethical standards and posed no ethical concerns.

Written informed consent was obtained from all participants for study participation and for audio recording and analysis. Participants were informed that participation was voluntary and that they could withdraw permission at any time without affecting their clinical care.

### Participants

Consecutive cisgender adult men who underwent Type III thyroplasty between August 2020 and February 2023 were screened. Inclusion criteria: age ≥ 18 years; assigned male at birth and identifying as male; no prior laryngeal surgery or neurologic disease; no structural vocal fold pathology on laryngoscopy/stroboscopy. Structural vocal fold pathology was defined a priori as macroscopic mucosal or cartilaginous lesions visible on endoscopy (e.g., nodules, polyps, cysts, sulcus vocalis, scarring, Reinke’s edema, contact granuloma, leukoplakia, malignant lesions, congenital webs). Accordingly, patients with secondary/structural etiologies of a high-pitched voice (e.g., sulcus vocalis or vocal fold scarring) were excluded a priori under the structural pathology criterion. A formal diagnosis of mutational falsetto was not required for inclusion. Instead, surgical indication was primarily patient driven and based on the individual’s perceived functional, social, or psychosocial burden related to a persistently high-pitched voice within a shared decision-making framework.

Exclusion criteria: active dysphonia unrelated to pitch; postoperative complications precluding assessment; clinically suspected significant hearing loss; neurologic disease (including vocal fold paresis/paralysis); and incomplete postoperative recording. Hearing was screened clinically using a bedside whispered-voice test performed by the attending laryngologist at the preoperative clinic visit and corroborated as needed by medical-record review and patient report. Reliance on hearing devices at intake was considered exclusionary when likely to interfere with everyday communication or study tasks. Formal audiometry was not performed as part of this protocol, although the whispered voice test has acceptable diagnostic accuracy for detecting hearing impairment [14].

Preoperative sustained-vowel recordings were obtained, and all surgeries occurred within 30 days of the preoperative recording. The prespecified primary endpoint was a single postoperative voice recording intended to isolate the procedure-attributable effect of Type III thyroplasty. Postoperative timing varied across participants and is reported descriptively as median 254 days (IQR 182–344; range 42–932). Participants were contacted by phone/text to obtain the postoperative recording. When a recording was not obtained, reasons were prospectively documented, no response, scheduling or travel constraints, declining to return or withdrawal of permission.

Surgical candidacy was determined a priori using three prespecified components: functional burden of a persistently high-pitched voice on everyday communication and self-perception; anatomic suitability on laryngoscopy/stroboscopy in the absence of structural vocal-fold pathology; and a shared decision-making process. During counseling, alternatives (watchful waiting, therapy, or surgery), expected benefits, and risks were reviewed, and the patient elected surgery with informed consent.

This framework aligns with the physiologic rationale of Type III thyroplasty, which lowers pitch by relaxing the anterior commissure thyroid cartilage complex, producing a more relaxed vocal fold configuration and reduced F0. It also accords with clinical series reporting benefit among patients who experience misgendering or body-image conflict, as well as frequent misperception in telephone or nonvisual contexts due to a persistently high-pitched voice [7].

Consistent with this literature, surgical candidacy was not based on an absolute preoperative F0 threshold. Patients with relatively low baseline F0 but substantial pitch related burden were also considered eligible. Early physiologic investigations and subsequent case series likewise describe significant pitch lowering and improved perceptual and psychosocial outcomes following Type III thyroplasty, supporting an indication grounded in symptoms and patient goals rather than a single numeric cutoff [9, 11]. 

**Fig. 1:**
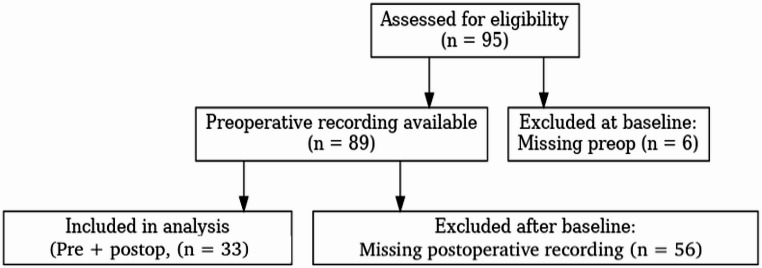
STROBE-style flow of participants: assessed (*n* = 95), preoperative recording available (*n* = 89), included in analysis (pre + postoperative recording, *n* = 33), and excluded after baseline (missing postoperative recording, *n* = 56)

Of 95 patients assessed, 89 had a preoperative recording; 33 provided both preoperative and postoperative recordings and were included in the analysis (Fig. 1). The analytic cohort’s age range was 23–58 years (mean 34.97, SD 8.64).

### Surgical procedure

All procedures were performed by a single otolaryngologist under local anesthesia with light sedation to allow intraoperative phonation and voice assessment. An anterior midline rectangular thyroid cartilage window was created using Isshiki Type III relaxation technique. Intermittent flexible nasolaryngoscopy was used as needed to visualize the larynx during window creation and adjustment, complemented by real-time auditory monitoring of the patient’s voice during phonation. The anterior thyroid lamina was posteriorly displaced to reduce longitudinal vocal fold tension and secured with non-absorbable 3 − 0 polypropylene (Prolene) sutures (no implant material was used). Mean operative time was approximately 45 min. Postoperatively, patients received 5 days of oral antibiotics and NSAIDs as needed; voice rest was not prescribed. To isolate the surgical effect, no structured pre- or postoperative voice therapy was delivered. 

### Acoustic recording

All pre- and postoperative recordings were obtained by the first author (speech-language pathologist) using a standardized protocol. The operating otolaryngologist (third author) performed all surgeries but had no role in acoustic data acquisition or measurement. Prior to acoustic analysis, recordings were de-identified and assigned randomly generated study codes by the second author. F0 extraction in Praat was performed by the first author blinded to recording time point (preoperative vs. postoperative), using the prespecified 3-s mid-segment procedure. Recordings were obtained preoperatively and at a single postoperative time point; surgeries occurred within 30 days after the preoperative recording, and postoperative timing varied across participants (Table 2) in a quiet clinic room using a standardized sustained-vowel protocol. iPhone 11 Pro units (iOS 14+) with the native Voice Memos app were used. The device was positioned 10 cm from the mouth at a 90° angle, following Titze and Winholtz [15]. Files were captured as M4A and converted to WAV 44.1 kHz using Audacity. Smartphone recordings provide accurate F0 under standardized placement [16, 17].

To isolate the pitch-specific surgical effect and minimize measurement noise with smartphone capture, we did not compute perturbation measures (e.g., jitter, shimmer), which are highly sensitive to phonatory intensity and environmental variability; instead, analysis was restricted to F0 from sustained vowels [18, 19]. Although participants also produced brief connected-speech samples (counting 1–10 and briefly describing how they felt), only sustained vowel segments were used for acoustic analysis in this study. This decision was based on previous literature indicating that sustained vowels yield more stable F0 measurements [16, 17].

### Acoustic analysis

Audio was imported into Praat (v6.2.01) [20] for analysis. From each token, a 3-s mid-segment of sustained/a/was manually extracted. The primary parameter was mean F0 (Hz) computed over this segment. Data were exported to a spreadsheet for statistical analysis. This smartphone-based protocol has been validated for accurate F0 estimation and yields results comparable to laboratory microphones, including in nonoptimized clinical settings [17, 21]. Notably, reported smartphone–clinical recording system discrepancies in the recent literature primarily involve voice-quality measures (e.g., HNR, AVQI, jitter) rather than F0, which is our sole acoustic endpoint [22].

### Statistical analysis

Data were analyzed using IBM SPSS Statistics v25. The distribution of the data was assessed using the Kolmogorov–Smirnov test. Since the data were normally distributed, a paired-samples t-test was performed to compare pre- and postoperative F0 values. We report the mean paired difference (ΔF0 = post – pre) with 95% confidence interval and Cohen’s dz as the effect size. Two-tailed α = 0.05 defined statistical significance. Descriptive statistics include mean, SD, median, and range. To evaluate potential time confounding, we secondarily modeled ΔF0 as a function of postoperative days using simple linear regression.

## Results

Thirty-three cisgender men (*N* = 33) completed both assessments (Fig. 1). Of the 89 patients with a preoperative recording, 33 (37.1%) provided a postoperative recording, while 56 (62.9%) were lost to follow-up (Fig. 1). To assess potential selection bias due to loss to follow-up, we compared baseline preoperative F0 between participants who provided a postoperative recording (*n* = 33) and those lost to follow-up (*n* = 56). Baseline preoperative F0 was comparable between groups (123.32 ± 23.76 Hz vs. 126.04 ± 20.52 Hz; mean difference − 2.72 Hz (95% CI − 12.65 to 7.21), suggesting no evidence of baseline pitch–driven selection. Documented reasons for missing postoperative recordings were predominantly practical/nonmedical (e.g., no response to calls or messages, scheduling or travel constraints). A subset withdrew permission to provide postoperative voice samples. Baseline and postoperative F0 summary statistics are shown in Table 1. Postoperative timing varied across participants (median 254 days; IQR 182–344; range 42–932; Table 2).

**Table 1:** Pre- and postoperative F0 in the analytic cohort (*N* = 33)

Measurements	Preoperative		Postoperative		ΔF0 (post–pre) mean (95% CI)	t	*p*	dz
	Mean ± SD	M (Min - Max)	Mean ± SD	M (Min - Max)				
**Mean F0 (Hz)**	123.32 ± 23.76	121.43 (86.14–183.52.14.52)	107.58 ± 19.15	101.75 (79.97–152.80)	−15.74 [−23.72,−7.76]	*4.002*	*< 0.001*	*0.70*

Mean average, *M* Median, *SD* standard deviation, Δ*F0* post – pre (negative indicates a decrease). 95% CI refers to the mean paired difference. dz = paired effect size (t/√n)

**Table 2:** Postoperative timing of the analytic cohort (*N* = 33). Surgeries occurred within 30 days after the preoperative recording; timing is reported descriptively (median, IQR, range)

*N*	Median days	IQR days	Range days	Median weeks	IQR weeks	Range weeks
33	254	182–344	42–932	36.3	26.0–49.1	6.0–133.1

*IQR* interquartile range (25th–75th percentiles; shown as Q1–Q3). Days reflect the elapsed interval between pre- and post-recordings (proxy for postoperative timing); surgeries occurred within 30 days after the preoperative recording. Range denotes minimum and maximum. No imputation was performed; all 33 cases with complete pre/post recordings and dates were included

**Fig. 2:**
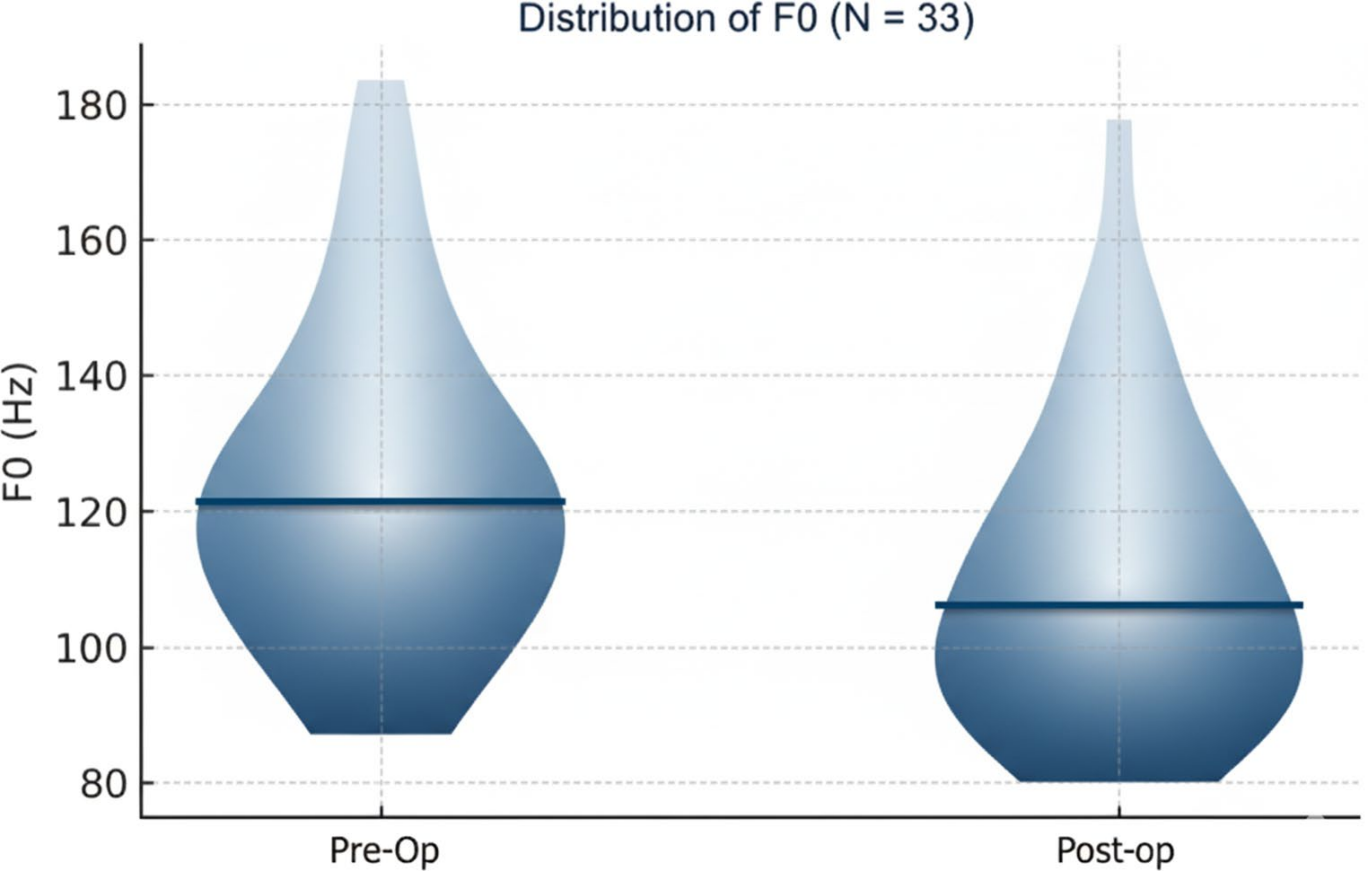
Distributions of F0 on sustained/a/preoperatively and postoperatively (*N* = 33). Violin plots display kernel density; solid lines indicate medians (dashed lines, quartiles). Group mean decreased from 123.32 Hz to 107.58 Hz (ΔF0 = − 15.74 Hz)

Mean F0 decreased from 123.32 Hz (SD 23.76) preoperatively to 107.58 Hz (SD 19.15) postoperatively; the paired mean difference was ΔF0 = − 15.74 Hz, t (32) = 4.002, *p* < 0.001, 95% CI − 23.72 to − 7.76, dz = 0.70. (Figure 2)

Individual changes in F0 were heterogeneous. Four participants (4/33, 12.1%) showed no F0 lowering (ΔF0 ≥ 0 Hz). Across the cohort, absolute magnitudes of F0 change were distributed as follows: <10 Hz in 10/33 (30.3%), 10–<20 Hz in 6/33 (18.2%), 20–<30 Hz in 8/33 (24.2%), 30–<40 Hz in 3/33 (9.1%), and ≥ 40 Hz in 2/33 (6.1%). In relative terms, the mean percentage reduction in F0 was 11.20% (SD 14.73%; 95% CI 5.98% to 16.42%). When stratified by baseline preoperative F0, reductions were larger among participants with baseline F0 > 140 Hz (*n* = 8; 38.57 ± 28.69 Hz; 23.83% ± 15.57%). Corresponding reductions were smaller in the 100–<140 Hz group (*n* = 21; 8.90 ± 15.56 Hz; 7.41% ± 13.20%) and the < 100 Hz group (*n* = 4; 5.41 ± 5.22 Hz; 5.82% ± 5.39%); these subgroup results are descriptive given small sample sizes (Table 3).

**Table 3:** Distribution of individual absolute F0 changes

Absolute F0 reduction category (Hz)	*n*	%
No F0 lowering (ΔF0 ≥ 0 Hz)	4	12.1
< 10 Hz reduction	10	30.3
10–<20 Hz reduction	6	18.2
20–<30 Hz reduction	8	24.2
30–<40 Hz reduction	3	9.1
≥ 40 Hz reduction	2	6.1

ΔF0 = post − pre; negative values indicate F0 lowering. Interval categories are based on absolute magnitudes of F0 lowering; “no lowering” indicates ΔF0 ≥ 0

**Fig. 3:**
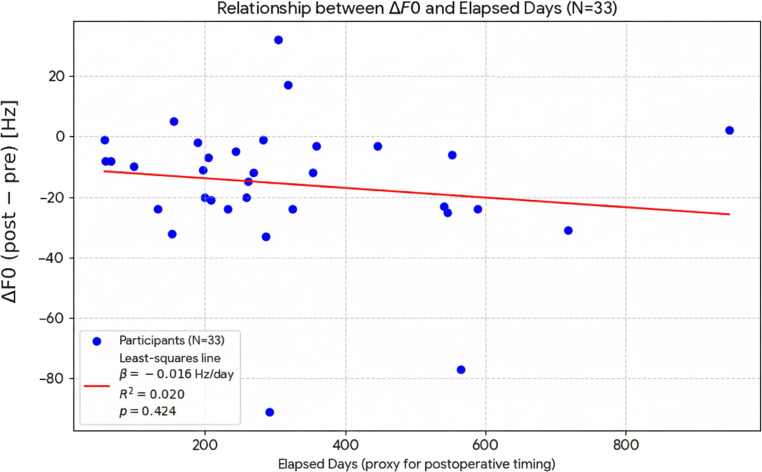
Relationship between ΔF0 (post − pre) and elapsed days between pre- and post-recordings (proxy for postoperative timing), *N* = 33. A least-squares line is shown; ΔF0 was not associated with elapsed days (β = −0.016 Hz/day, *p* = 0.424, R² = 0.020)

No intra- or postoperative complications were recorded in the analytic cohort. In a sensitivity analysis, ΔF0 was not associated with postoperative days (β = −0.016 Hz/day, *p* = 0.424, R² = 0.020) (Fig. 3).

## Discussion

In this prospective single-center cohort of cisgender men with persistently elevated pitch, Type III thyroplasty produced a moderate reduction in mean F0 (ΔF0 = − 15.7 Hz; dz = 0.70), lowering group mean values from 123.3 to 107.6 Hz at a single postoperative recording obtained a median of 254 days after surgery. Expressed musically, this corresponds to roughly a 2–3 semitone decrease, a difference that is readily perceptible in routine clinical practice. Although the cohort’s mean preoperative F0 was not markedly elevated, all participants reported meaningful functional or social distress from their high-pitched voice; thus the indication for surgery reflected symptom burden and patient preference rather than an absolute numeric F0 threshold. Consistent with this rationale, surgical decision-making in high-pitch complaints typically considers functional and psychosocial impact alongside acoustic measures [7, 12, 23]. Within the observed range of follow-up times, the magnitude of F0 change was not associated with postoperative timing (β = −0.016 Hz/day, *p* = 0.424; Fig. 3), suggesting that the reduction we observed reflects a stable procedure-related shift rather than an artifact of when the postoperative recording was obtained.

The magnitude of F0 reduction observed in our cohort was more modest than that reported in some prior series and pooled estimates, particularly in cohorts enriched for classic mutational falsetto/puberphonia with substantially elevated baseline F0 [12, 24]. In a frequently cited single-center series, mean F0 decreased from 187 Hz to 104 Hz, and a recent meta-analytic pooled estimate reported a mean reduction of − 75.9 Hz, with larger decreases at higher baseline F0 [12, 24]. A plausible explanation in our study is case mix and baseline pitch distribution: a formal diagnosis of mutational falsetto was not required, and a substantial proportion of participants entered surgery with baseline F0 values closer to the typical adult male speaking range, limiting the achievable absolute reduction (ceiling effect). Accordingly, indications in our cohort reflect broader, symptom-driven use of Type III thyroplasty grounded in patient-reported functional, social, and psychosocial burden rather than strict selection by a single acoustic cutoff. While motivation was not formally quantified, we do not interpret these findings as a purely “aesthetic” shift; instead, they support shared decision-making and reinforce the importance of counseling patients about baseline-dependent variability in expected effect size.

Our findings corroborate prior reports showing clinically relevant pitch lowering after Type III thyroplasty across a variety of indications [8–10, 13, 24–26]. Early case series and small cohorts, particularly in mutational falsetto or very high baseline populations, reported larger absolute F0 drops (e.g., 150–245 Hz reduced toward 98–141 Hz) [8, 9, 24, 26]. Evidence synthesized across studies likewise indicates an overall decrease following thyroplasty, albeit with between-study heterogeneity related to diagnosis and technique [12]. Compared with these reports, the present study contributes a larger homogeneous cis-male cohort (*N* = 33) assessed with a standardized recording protocol and a prespecified, pitch-focused primary endpoint, thereby offering pragmatic benchmarks for postoperative counseling across real-world follow-up intervals.

The comparatively low baseline F0 in our cohort (123 Hz) is likely explained by several design choices. First, in contrast to series enriched for mutational falsetto or gender-affirming cases with markedly elevated starting pitch, our sample included cisgender men only, which naturally yields lower preoperative F0 and smaller absolute ΔF0. For example, a recent retrospective study in trans men reported a mean reduction from 156 to 109 Hz at 6 months, a change largely driven by their higher baseline pitch [23]. Second, we derived F0 from a 3-s mid-segment of sustained/a/recorded with a fixed 10-cm mouth-to-microphone geometry on a smartphone device. This protocol was chosen to maximize F0 stability and is supported by contemporary validation studies showing that, under standardized placement, smartphone recordings provide acoustic measurements comparable to laboratory “gold-standard” systems [21]. Finally, we deliberately restricted our acoustic endpoint to F0. Perturbation-based metrics and patient-reported outcomes, although clinically important, are highly sensitive to phonatory intensity and environmental conditions and may be less reliable when collected once in variable follow-up using smartphones [18, 19]. By focusing on a single, mechanistically proximal outcome, this study prioritizes internal validity; multidimensional acoustic, perceptual, and quality-of-life outcomes are planned for subsequent longitudinal cohorts.

Although most individuals demonstrated a reduction in F0, a minority showed minimal or paradoxical change, a pattern also described in earlier work [12, 21, 23]. Possible explanations include differences in baseline anatomy and phonatory behavior, the extent of cartilage repositioning, healing trajectory, and measurement variability inherent to single time point assessments. Such heterogeneity highlights the need for individualized postoperative follow-up and, when indicated, targeted voice therapy to optimize functional outcomes.

### Strengths and limitations

Strengths include the prospective single-center design, use of a single surgeon which reduces surgical variability, and a harmonized smartphone-based recording protocol supported by validation studies for acoustic accuracy in non-optimized clinical environments [15–17, 21, 22]. Consecutive recruitment, explicit inclusion and exclusion criteria, and transparent reporting of attrition and follow-up timing further strengthen internal validity.

Limitations include the focus on a single acoustic endpoint (F0 from sustained vowels) without connected-speech acoustics or patient-reported outcomes, and the absence of systematic diagnostic subtyping and detailed surgical parameters, which precludes subgroup inferences. We did not keep a study specific screening log for patients excluded due to secondary/structural etiologies (e.g., sulcus or scarring); therefore, no comparative data are available, and our findings apply to patients without macroscopic structural vocal fold pathology. The decision to restrict analysis to F0 reflects a deliberate mechanistic focus; however, future work should incorporate multidimensional acoustic, perceptual, and quality-of-life measures to contextualize pitch changes within functional voice outcomes. Despite attrition from screening to analysis, our analytic cohort (*N* = 33) compares favorably with sample sizes typically reported in single-center Type III thyroplasty series, though larger multicenter cohorts are still needed. Given loss to follow-up, baseline preoperative F0 was similar between completers and non-completers; however, differential attrition related to unmeasured postoperative satisfaction cannot be excluded. Another methodological consideration is that only a single postoperative recording was obtained per participant, and the elapsed time between pre- and postoperative recordings varied (median 254 days, IQR 182–344). This pattern reflects real-world follow-up in our clinic and allowed us to quantify pitch change over the interval when most patients returned. In sensitivity analyses, F0 was not associated with elapsed days, suggesting that timing variability did not materially influence the observed pitch reduction; nevertheless, more frequent standardized follow-up would better characterize individual recovery trajectories. Future multicenter studies incorporating repeated recordings at predefined time points (e.g., 3 and 12 months), along with detailed surgical parameters, connected-speech measures, and patient-reported outcomes, could further refine benchmarks and help identify which patients experience the greatest and most durable benefit.

## Conclusions

Type III thyroplasty yields a clinically interpretable reduction in F0 in cisgender men when measured under a standardized protocol and in the absence of structured perioperative voice therapy. These findings provide pragmatic reference values for interpreting postoperative F0 change in similar clinical settings and can support postoperative assessment, counseling, and shared decision-making.

## Data Availability

De-identified data are available from the corresponding author on reasonable request.
